# Sex Determination in Man

**Published:** 1917-09-01

**Authors:** 


### THE INSTITUTIONAL LIBRARY.

### SEX DETERMINATION IN MAN.

To have reached a genuine second edition is a distinction for any book, and especially perhaps for a medical one. Mr. Rumley Dawson's volume on the causation of sex has earned this distinction, and therefore a little space may be well spent in examining what he has to say on this most important subject, upon which, unless humanity manages in future to settle its differences without a ruinous expenditure of male life, may even depend the continuation of the white races. The first thing to note is that Mr. Dawson's theory is simple and definite, which is what a theory should be. A vague, long-drawn-out hypothesis, involving a great deal of new terminology, and framed to include somehow or another ill-connected facts, nearly always succumbs pretty soon to the sharp edge of the razor of Occam. This is one of the few points of resemblance between science and art, that a good theory is like a good plot. If there *is* a plot, critics say, a child could tell it. And similarly, most of the verified theories and of the successful inventions seem, once they are out, as though a child could have thought of them. "The child-like faculty that divines"—a great deal could be said as to that; but the claims of the matter in hand impel to substitution of the statement that we are not hailing Mr. Dawson as the solver of the riddle of sex. He is merely given credit here for the uncommon achievement of original thinking successfully communicated.

What he maintains is that the sex of the child is already determined in the unimpregnated ovum, the right ovary furnishing males and the left one females; that the ovaries ovulate alternately; so that if the sex and date of birth of the eldest child are known, also the number of menstruations preceding fertilisation, the sex of all subsequent children can be predicted, or even determined, with a great measure of success. The author claims for himself success to the amount of 97 per cent. Now the verification of this idea is not so good as its form and stating. Obviously evidence in support of such a definite and wide-reaching novel statement on such an everyday subject must be drawn from many fields of study, and must be closely tested before being put forward. There is a slightly popular tone about the book which is not too reassuring. But we have not yet seen a fatal criticism of the writer's contentions, or, at least, one against which defence is not made in his twenty-first chapter. The planning of his subject-matter is good—literature of the subject, the new hypothesis, evidence indirect, evidence direct, objections, and so forth—but it all wants to be rather better done, except the generalisation itself, the pith of the matter. So let not Mr. Dawson be cast down by the criticism of the more erudite: if he is right, his name will be known a century hence, which is more than can be said for one in ten thousand of the compilers of orthodox text-books, *bien documentés*.

Meanwhile, there are other new Richmonds in the field. The Mendelians, whose results, like medical laboratory experiments, are so admirable with lower forms of life, but somewhat fallacious as applied to man, have not made much headway with their arguments on sex causation. Lately, in Germany, one or two authors have put forward the statement that the matter is closely concerned with the time relation of impregnation to menstruation. Women impregnated within ten days after the cessation of the menses produce a high percentage of boys, and are, too, much more susceptible of fertilisation then. Conversely from the tenth to the twenty-first day girls are more numerous, whilst in the week immediately preceding menstruation there is almost a physiological sterility. It would be interesting to have Mr. Dawson's views upon this theory. This war, as has been said, lends especial interest to the subject of sex determination in man, but the interest is not an immediate one. In the rising generation the proportions of the sexes are happily unaffected. Nevertheless, the revelation of what war, with its combination of high explosives and low birth-rates, will mean to civilised nations in future would be less disquieting if the aspiring will of humanity to divinity could be gratified by the attainment of some control of the sex constitution of its populations. At present it looks, too, as if many social problems could be rendered less knotty by such increase of knowledge and power, dangerous though that increase might be. Growing industrialisation, for instance, has meant up till now a male mortality still further surpassing the female one. The phenomenon of the mateless woman, which is at the bottom of the female suffrage movement, seems one not easy of removal by measures now possible.

### THE RUHLEBEN CAMP MAGAZINE.

APPARENTLY the regulations of the camp for British civilians interned in Ruhleben are not so strict as to preclude the publication of a camp magazine in English. Since this publication is little known in England, we think that our readers will be interested in hearing a description of its contents, as represented by the June number, which lies before us. This is the sixth number of the series, which is published at the price of 50 pfennigs per copy. What is the extent of its circulation and how far it is read outside the camp does not appear, though we learn that nearly 7,000 copies of the Christmas Number were sold, of which more than half went to foreign countries. Indeed, one of the interests of the magazine is the number of questions which it raises, and does not answer. Although the magazine runs to seventy-two pages, and is profuse in illustrations, very little more than a general idea can be gleaned from it as to the prisoners' condition and manner of life. Under sentence of perpetual holiday, the prisoners have done their best to alleviate the boredom of their lot, and have undertaken courses of study, from which they would probably have shrunk at any other time. Their most noteworthy achievement, news of which reached English newspapers on Sunday last, has been to pass at Ruhleben the London Matriculation Examination, which six members of the camp have done. The examination was held in Ruhleben in December, and the candidates took advantage of the educational classss which have been held there, thanks to the aid of the British Prisoners of War Book Scheme. By this means the necessary books were supplied, and the specially prepared examination papers were sent through the same agency. One of the successful candidates, Mr. L. K. White, of Edinburgh, wished also, as an Edinburgh man, to matriculate at that University, and new papers prepared by Edinburgh University were dispatched to Ruhleben. In these he was also successful, which probably constitutes a record for any interned prisoner of war.

The magazine is rich in illustrations and portraits, largely from the pencil of Mr. C. M. Horsfall. The camp possesses a school, with a nautical department, an engineering class, an orchestra, an arts and science union, and a theatre. Even a camp bulb show was held in April. News of these activities, which includes a reference to Mr. Morvaren's overture, to the camp's well-known production of "Androcles and the Lion" (the first original musical composition of the camp), fill the corners of the magazine. The rest is a riot of comic verse and comic articles. The verse is unusually good, and the whole standard of the magazine shows that there is no better stimulus to originality than a sentence to perpetual holiday. We only wish we had space to reproduce some of the drawings, though we are informed that the "articles and drawings are the property of the respective writers and artists, who have in every case reserved the copyright."

**The Pneumothorax Treatment of Pulmonary Tuberculosis.** By CLIVE RIVIÈRE, M.D., F.R.C.P. Oxford Medical Publications. (London: Henry Frowde, Hodder and Stoughton. 1917. Pp. xiii + 186.)

As was to be expected from its authorship and source, this not so very little book is an excellent transcript, from the point of view of ordinary practice, of the literature of the treatment of phthisis by artificial pneumothorax; that is, of the greater part of lung collapse therapy. Dr. Rivière has evidently worked hard at the subject, and thought over all that he has read on it. Where the book could be improved is in the introduction of practical points probably to be learnt only by actual work abroad or by tuition from those who have had that advantage. It would have been a good thing if Dr. Lillingston, the introducer, almost, of artificial pneumothorax work into England, had collaborated. The earliest case of his own that the author communicates was in 1914, whereas in Hamburg patients had been treated in 1905, in Scandinavia prior to 1909; while in 1907 Forlanini had got so far as to start his *Rivista delle pubblicazioni sul pneumotorace terapeutico*. The practical points we mean are such as the following. In anæsthetising the site of a puncture, the local anæsthetic must be dissolved in a very small quantity of fluid, not more than eight minims; otherwise there is risk of the after-coming pneumothorax needle getting wet inside and not transmitting oscillations well. Saugman has even proposed dispensing with anæsthetisation on this account. The "snap" announcing penetration of parietal pleura may be absent, and yet a good pneumothorax be secured. As regards manometric readings a great deal more information should have been given as to the difference between pleural and pulmonary oscillations, the all-important guide as to whether the needle is in the right or the wrong place. "Scenting the gas" is not mentioned. Even with the cannula (which has sharp edges), recommended by the author, there is quite a possibility of wounding the lung. Again, the effect on the manometer of blood slowly rising in the needle should have been given. Finally, after every filling, the patient should be strictly kept from lying on the sound side, to avoid gravitation of sputum into the better lung. One could go on thus a little longer, but it is high time to stop, for in all reviewing the interdigitations of the author's and the critic's knowledge are very apt to be stated in a one-sided and incomplete fashion which is unfair to the former. A good manual, then; but it would have augured better for British medicine to have had instead a piece of research.

**The Secretion of the Urine.** By ARTHUR R. CUSHNY, M.D., F.R.S. (London: Longmans, Green and Co. 1917. Pp. 238 + xi. Price 9s. net.)

THIS is one of a series of "Monographs on Physiology" issued under the editorship of Professor Starling. The volumes generally are intended to be careful and complete studies of particular and limited aspects of human physiology, and each, in its own department, must be accepted as the latest expert word on the subject of which it treats. Such a statement is fairly applied to Professor Cushny's book, and no one can read the volume without being impressed both by the thoroughness of its plan and by the lucidity of its method. The complexity of the problem is known only to the initiated, but a measure of the range of the discussion may be found in the bibliography here supplied, for it numbers over four hundred references. Even when all is said that can be said it remains true that there are still dark places in our knowledge of the physiology of the kidney, and the physiologists have not fully succeeded in convincing one another. Professor Cushny does not pretend to deal with pathological disturbances of kidney function, but in many of his chapters there are discussions of direct interest to the practical physician, and he adds a chapter on nephritis and other renal disorders. We have much sympathy with his suggestion that the clinical observer ought at least in the meantime to stick to his own immediate field of observation, and to avert his gaze alike from the physiological laboratory and the *post-mortem* theatre.

